# Association of early severe thrombocytopenia and platelet course with in-hospital mortality in critically ill children

**DOI:** 10.3389/fped.2022.922674

**Published:** 2022-08-02

**Authors:** Yan Lu, Chaoxiang Ren, Haoyang Guo

**Affiliations:** Clinical Laboratory, DongYang People's Hospital, Dongyang, China

**Keywords:** severe thrombocytopenia, platelet course, in-hospital mortality, critically ill children, Pediatric Intensive Care (PIC) database

## Abstract

This study aimed to assess the association of early severe thrombocytopenia and platelet course with in-hospital mortality in critically ill children. Data of critically ill children in this study were obtained from the Pediatric Intensive Care Database. Patients with and without severe thrombocytopenia were adjusted for covariates using propensity score matching (PSM) to ensure the robustness of the results. Univariate and multivariate logistic regression analyses were performed on the original and PSM cohorts, respectively. Results are presented as odds ratios (ORs) with 95% confidence intervals (95% CI). In studies of the platelet course, logistic regression analysis was used to assess the effect of different degrees of recovery on in-hospital mortality in critically ill children with early severe thrombocytopenia. The study included 4,848 critically ill children, of whom 450 with early severe thrombocytopenia were matched to 450 without early severe thrombocytopenia. Univariate and multivariate logistic regression results showed that early severe thrombocytopenia was an independent risk factor for in-hospital mortality in critically ill children in both the original and PSM groups. In addition, the study results of platelet course showed that the recovery of platelet count to ≥150 × 10^9^/L in the short term was a protective factor for the prognosis of patients (OR, 0.301; 95% CI, 0.139–0.648, *P* = 0.002). Our study revealed that early severe thrombocytopenia is an independent risk factor for in-hospital mortality in critically ill children. In addition, in-hospital mortality was significantly reduced in children with early severe thrombocytopenia, whose platelet count returned to normal levels in the short term.

## Introduction

Early prognostic assessment of critically ill children is critical. If children with poor prognoses are identified earlier, the clinical interventions can be immediately implemented, resulting in a higher survival rate. However, children are less able to describe their own conditions than adults and find it more difficult to cooperate with some clinical examinations. Although intensive care for pediatric patients has greatly improved in recent decades, there are few independent, objective, and easily accessible early indicators of mortality in critically ill children.

Platelets serve various functions, including hemostasis, thrombosis, inflammation, and vascular regeneration (1). Platelet count is an objective indicator determined by routine blood tests. As a result, changes in absolute platelet count can be observed in a wide range of diseases involving various organ systems (2–4). A decrease in platelet count is common in critically ill patients (5, 6). The most common explanation is that platelets are activated to help with hemostasis, resulting in “consumption” exceeding “production” (7).

Patients with absolute platelet counts of <50 × 10^9^/L, as opposed to mild thrombocytopenia, frequently require clinical interventions, such as platelet transfusions, to prevent bleeding (8). Therefore, severe thrombocytopenia requires more medical attention. Severe thrombocytopenia is a prognostic risk factor in critically ill neonatal and adult patients, with increased mortality and hospital stay (9, 10). However, owing to a lack of relevant studies, the prognostic value of early severe thrombocytopenia in critically ill children remains unknown.

Therefore, we verified the prognostic value of early severe thrombocytopenia in critically ill children using a retrospective analysis of a large public database called Pediatric Intensive Care (PIC). This study aimed to investigate the association between early severe thrombocytopenia and platelet course and in-hospital mortality in critically ill children.

## Materials and Methods

### Data source

Pediatric Intensive Care is a pediatric-specific database that contains data on more than 13,000 admissions of critically ill pediatric patients admitted to the Children's Hospital of Zhejiang University School of Medicine between 2010 and 2018 (11). Children's Hospital of Zhejiang University School of Medicine is China's National Clinical Research Center for Children's Health. As a medical center exclusive to children, it contains 119 intensive care beds in cardiac, general, pediatric, surgical, and neonatal intensive care units (ICUs). Patient statistics of the PIC database stratified by care units can be found in the introduction to the original database (http://pic.nbscn.org) (11). Demographics, vital signs, laboratory measures, medications, and other clinical data were collected. This project was approved by the Institutional Review Board of the Children's Hospital affiliated with the Zhejiang University School of Medicine. As data were de-identified and retrospective research had no impact on clinical treatment, the requirement for patient consent was waived.

### Study population and data extraction

**Figure 1:**
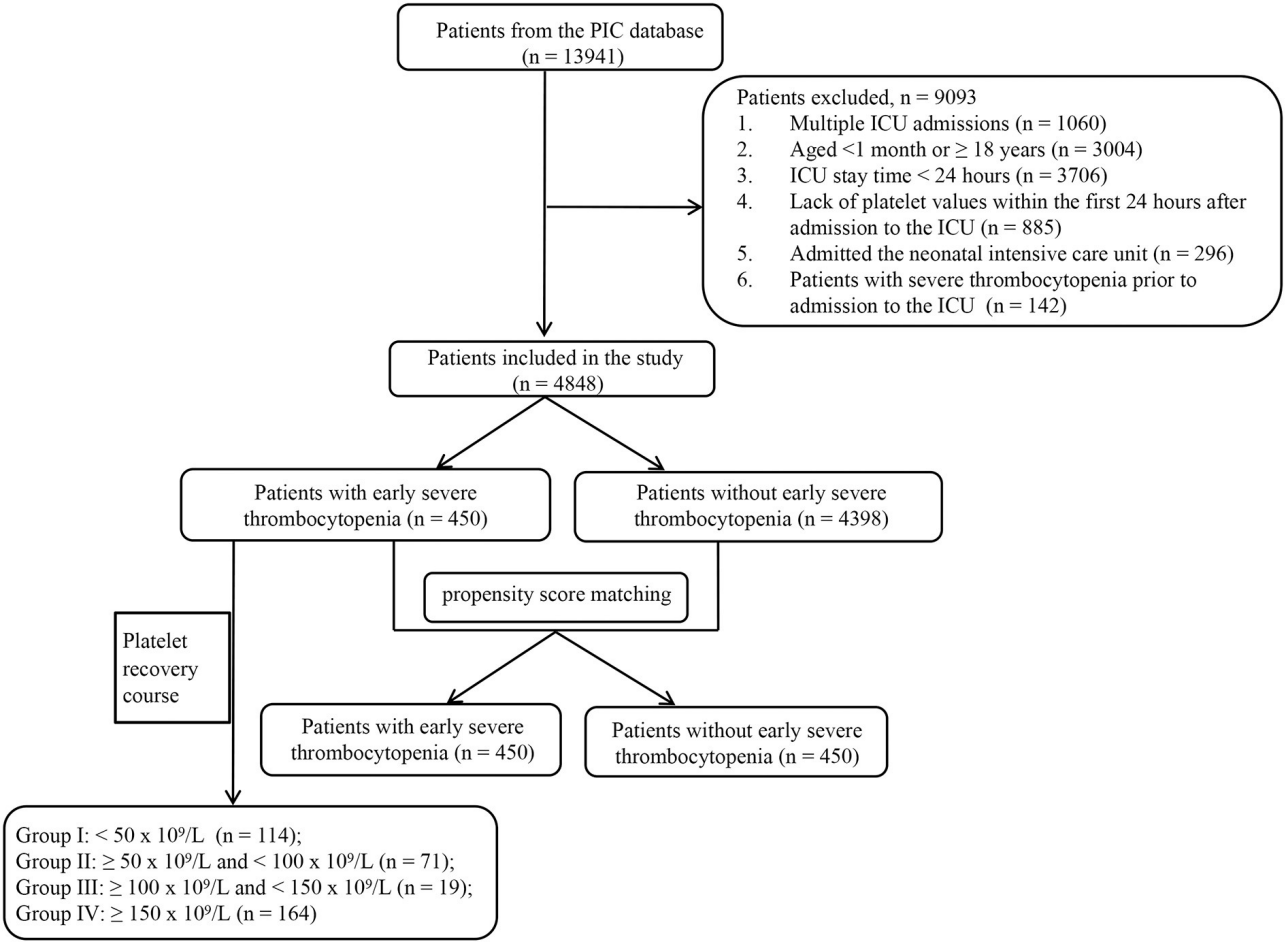
Flowchart for screening the study population.

Only patients hospitalized for the first time were included in this study. The exclusion criteria were as follows: (1) age <1 month or ≥18 years; (2) time in the ICU of <24 h; (3) lack of data on platelet counts within the first 24 h after admission to the ICU; (4) admission to the neonatal ICU; and (5) severe thrombocytopenia prior to admission to the ICU. Early severe thrombocytopenia was defined as a platelet count of <50 × 10^9^/L within the first 24 h of ICU admission. Patients with early thrombocytopenia who met one or more of the following criteria were included in the platelet course study: (1) patients whose maximum platelet count obtained within 72 h of ICU admission exceeded 150 × 10^9^/L after the minimum platelet count appeared in the first 24 h; (2) patients who had at least one platelet record every 24 h within 72 h of ICU admission. The patient's maximal platelet count within 72 h of ICU admission after the minimum platelet count within the first 24 h was considered short-term platelet recovery. Figure 1 illustrates the screening procedure.

The primary outcome of this study was all-cause mortality during the hospital stay. The secondary outcome was the length of the ICU stay. Data were collected using the PostgreSQL 10 software, including age, sex, first care unit, primary diagnosis on ICU admission, laboratory results, and length of ICU stay.

### Statistical analysis

Categorical variables were reported as numbers and percentages, and chi-square tests were used to assess differences between the groups. Continuous variables were reported as medians and interquartile distances, and Wilcoxon tests were used to assess differences between the groups. Stata software (version 14.0; Stata Corp., College Station, TX, USA) was used for all statistical analyses. *P* < 0.05 was considered statistically significant.

The primary diagnosis on ICU admission was extracted from the admission diagnosis according to the International Classification of Diseases 10 (ICD-10) code, and the underlying disease was determined if the patient was diagnosed with this disease category. A patient may have multiple diseases. Laboratory results were extracted from the worst results within 24 h after the patient entered the ICU.

To ensure the robustness of the study, propensity score matching (PSM) was used to adjust for age, sex, first care unit, and primary diagnosis on ICU admission. A 1:1 nearest-neighbor matching with a caliper width of 0 was applied for patients with early severe thrombocytopenia and without early severe thrombocytopenia. Univariate and multivariate logistic regression analyses were performed on the original and PSM cohorts, respectively. Results are presented as odds ratios (ORs) with 95% confidence intervals (95% CI).

In the study of the platelet course, patients were divided into four groups according to the short-term recovery of platelet counts (Group I: <50 × 10^9^/L; Group II: ≥50 × 10^9^/L and <100 × 10^9^/L; Group III: ≥100 × 10^9^/L and ≥150 × 10^9^/L; Group IV: ≥150 × 10^9^/L). A logistic regression analysis was performed to investigate the influence of varying degrees of recovery on in-hospital mortality in patients with early severe thrombocytopenia.

## Results

### Patient characteristics in the original cohort

The study included data from 4,848 critically ill children. Early severe thrombocytopenia occurred in 450 patients, with an incidence rate of 9.3%. Table 1 shows the baseline characteristics of patients with and without early severe thrombocytopenia in the original cohort. The number of patients with and without early severe thrombocytopenia was significantly different in the first care unit. Regarding the primary diagnosis upon ICU admission, we found that patients with early severe thrombocytopenia were more likely to develop neoplasms, diseases of the circulatory system, and sepsis compared to patients without early severe thrombocytopenia. In addition, patients with early severe thrombocytopenia showed a higher maximum value of pH, creatinine, serum potassium, prothrombin time, and partial prothrombin time, and exhibited lower minimum partial pressure of oxygen and white blood cells.

**Table 1:** Patient characteristics in the original cohort and the PSM cohort.

**Variables**	**Original group**			**PSM group**		
	**Early severe thrombocytopenia (*n* = 450)**	**Without early severe thrombocytopenia (*n* = 4,398)**	***P*-value**	**Early severe thrombocytopenia (*n* = 450)**	**Without early severe thrombocytopenia (*n* = 450)**	***P*-value**
**Age**, ***n*** **(%)**
<12 months	180 (40.0)	1,794 (40.8)	0.473	180 (40.0)	184 (40.9)	0.955
≥12 and <36 months	101 (22.4)	1,070 (24.3)		101 (22.4)	98 (21.8)	
≥36 and <216 months	169 (37.6)	1,534 (34.9)		169 (37.6)	168 (37.3)	
**Sex**, ***n*** **(%)**
Male	260 (57.8)	2,471 (56.2)	0.516	260 (57.8)	270 (60.0)	0.498
Female	190 (42.2)	1,927 (43.8)		190 (42.2)	180 (40.0)	
**First care unit**, ***n*** **(%)**
Cardiac intensive care unit	40 (8.9)	1,315 (29.9)	<0.001	40 (8.9)	41 (9.1)	0.981
General intensive care unit	186 (41.3)	921 (20.9)		186 (41.3)	185 (41.1)	
Pediatric intensive care unit	194 (43.1)	1,306 (29.7)		194 (43.1)	191 (42.4)	
Surgical intensive care unit	30 (6.7)	856 (19.5)		30 (6.7)	33 (7.3)	
**Primary diagnosis on ICU admission**, ***n*** **(%)**
Neoplasms	56 (12.4)	87 (2.0)	<0.001	56 (12.4)	57 (12.7)	0.920
Diseases of the respiratory system	66 (14.7)	659 (15.0)	0.857	66 (14.7)	65 (14.4)	0.925
Diseases of the genitourinary system	5 (1.1)	64 (1.5)	0.557	5 (1.1)	4 (0.9)	0.738
Diseases of the circulatory system	22 (4.9)	478 (10.9)	<0.001	22 (4.9)	22 (4.9)	1.000
Diseases of the nervous system	38 (8.4)	468 (10.6)	0.147	38 (8.4)	38 (8.4)	1.000
Diseases of the digestive system	23 (5.1)	229 (5.2)	0.931	23 (5.1)	24 (5.3)	0.881
Sepsis	34 (7.6)	120 (2.7)	<0.001	34 (7.6)	33 (7.3)	0.899
**Laboratory results**
PH_max	7.50 (7.46–7.55)	7.50 (7.46–7.54)	0.017	7.50 (7.46–7.55)	7.50 (7.46–7.54)	0.151
PCO_2__max (mmHg)	48.55 (39.9–63)	47.5 (42.1–56.2)	0.737	48.55 (39.9–63)	47.05 (40.3–58.2)	0.270
PO_2__min (mmHg)	42 (32–67.9)	46.8 (35.6–72.6)	<0.001	42 (32–67.9)	43.45 (33.7–66.4)	0.232
Creatinine_max (μmol/L)	50 (40–72)	47 (41–57)	<0.001	50 (40–72)	47 (40–59.4)	0.011
Potassium_max (mmol/L)	4.7 (4.3–5.5)	4.5 (4.2–5)	<0.001	4.7 (4.3–5.5)	4.6 (4.3–5.1)	0.061
Glucose_max (mmol/L)	11.4 (8.1–17.8)	11.4 (8.7–15.1)	0.397	11.4 (8.1–17.8)	10.95 (8.3–15.3)	0.236
PT_max (s)	14.9 (12.8–20.2)	14.1 (12.4–16.4)	<0.001	14.9 (12.8–20.2)	13.6 (11.9–16.5)	<0.001
PTT_max (s)	42.35 (33.3–72)	37.2 (30.7–49.9)	<0.001	42.35 (33.3–72)	37.2 (29.7–53.1)	<0.001
WBC_min (10^9^/L)	3.59 (1.01–5.86)	6 (4.26–7.78)	<0.001	3.59 (1.01–5.86)	5.51 (3.23–7.81)	<0.001
**Outcome**
In-hospital mortality, *n* (%)	71 (15.8)	280 (6.4)	<0.001	71 (15.8)	39 (8.7)	0.001
ICU LOS, days	5.73 (2.79–12.13)	3.88 (1.96–7.92)	<0.001	5.73 (2.79–12.13)	5.54 (2.79–10.88)	0.633

*ICU, intensive care unit; PCO_2_, partial pressure of carbon dioxide; PO_2_, partial pressure of oxygen; PT, prothrombin time; PTT, partial thromboplastin time; WBC, white blood cell; LOS, length of stay*.

The in-hospital mortality rate of patients with early severe thrombocytopenia was 15.8%, which was significantly higher than that of patients without early severe thrombocytopenia (*P* <0.001). In addition, patients with early severe thrombocytopenia had significantly longer ICU stays than non-patients [5.73 (2.79–12.13) vs. 3.88 (1.96–7.92) days; *P* < 0.001].

### Patient characteristics in the PSM cohort

Age, sex, primary care unit, and underlying conditions were considered in the PSM analysis. A total of 450 patients with early severe thrombocytopenia were matched with 450 patients without early severe thrombocytopenia. In the PSM cohort, patients with early severe thrombocytopenia had a higher in-hospital mortality rate than those without early severe thrombocytopenia (15.8% vs. 8.7%; *P* = 0.001). However, the length of ICU stay did not differ between the two groups (Table 1).

### Early severe thrombocytopenia and in-hospital mortality

In the original cohort, using univariate logistic regression, early severe thrombocytopenia, sex, first care unit, neoplasms, diseases of the respiratory system, sepsis, maximum partial pressure of carbon dioxide, minimum partial pressure of oxygen, maximum value of potassium, maximum value of prothrombin time, and maximum value of partial prothrombin time were considered statistically significant. Multivariate logistic regression analysis demonstrated that early severe thrombocytopenia was an independent risk factor for in-hospital mortality (Table 2).

**Table 2:** Univariate and multivariate logistic regression analysis to assess the association between early severe thrombocytopenia and in-hospital mortality in original group and PSM group.

	**Original group (*****n*** = **4,848)**				**PSM group (*****n*** = **900)**			
	**Univariate analysis**		**Multivariate analysis**		**Univariate analysis**		**Multivariate analysis**	
	**OR (95% CI)**	***P*-value**	**OR (95% CI)**	***P*-value**	**OR (95% CI)**	***P*-value**	**OR (95% CI)**	***P*-value**
Early severe thrombocytopenia	2.76 (2.08–3.65)	<0.001	1.69 (1.20–2.38)	0.002	1.97 (1.30–2.99)	0.001	1.67 (1.04–2.69)	0.034
Age	0.91 (0.80–1.03)	0.136	1.02 (0.88–1.18)	0.758	1.07 (0.86–1.35)	0.541	1.17 (0.88–1.54)	0.282
Sex	1.35 (1.08–1.68)	0.010	1.28 (1.00–1.65)	0.052	1.37 (0.90–2.09)	0.136	1.52 (0.93–2.45)	0.093
First care unit	1.47 (1.32–1.64)	<0.001	1.30 (1.15–1.48)	<0.001	1.62 (1.32–1.99)	<0.001	1.48 (1.15–1.90)	0.002
Neoplasms	2.99 (1.93–4.65)	<0.001	1.80 (1.02–3.16)	0.042	2.06 (1.24–3.42)	0.006	1.37 (0.68–2.75)	0.384
Diseases of the respiratory system	1.58 (1.21–2.07)	0.001	0.77 (0.54–1.08)	0.132	1.08 (0.62–1.89)	0.775	0.84 (0.40–1.76)	0.652
Diseases of the genitourinary system	0.58 (0.18–1.85)	0.356	0.35 (0.09–1.38)	0.134	2.07 (0.42–10.10)	0.368	2.20 (0.31–15.8)	0.434
Diseases of the circulatory system	0.96 (0.67–1.38)	0.827	1.14 (0.77–1.70)	0.505	1.38 (0.60–3.18)	0.446	1.69 (0.66–4.35)	0.276
Diseases of the nervous system	1.05 (0.74–1.48)	0.805	1.40 (0.94–2.08)	0.102	0.71 (0.32–1.59)	0.404	1.25 (0.50–3.11)	0.636
Diseases of the digestive system	0.69 (0.39–1.21)	0.193	0.79 (0.41–1.50)	0.466	0.85 (0.33–2.19)	0.734	1.19 (0.40–3.57)	0.752
Sepsis	2.09 (1.30–3.35)	0.002	1.72 (1.00–2.97)	0.051	2.24 (1.21–4.14)	0.010	2.66 (1.25–5.66)	0.011
PH_max	0.20 (0.04–1.09)	0.063	1.00 (1.00–1.00)	0.990	1.00 (1.00-1.00)	0.988	1.00 (1.00–1.00)	0.990
PCO_2__max	1.03 (1.03–1.04)	<0.001	1.03 (1.03–1.03)	<0.001	1.04 (1.03–1.04)	<0.001	1.03 (1.02–1.04)	<0.001
PO_2__min	0.98 (0.97–0.98)	<0.001	0.99 (0.99–1.00)	0.017	0.97 (0.96–0.98)	<0.001	0.99 (0.98–1.00)	0.286
Creatinine_max	1.00 (1.00–1.00)	0.062	1.00 (1.00–1.00)	0.721	1.00 (1.00–1.00)	0.578	1.00 (1.00–1.00)	0.683
Potassium_max	1.23 (1.18–1.29)	<0.001	1.09 (1.03–1.15)	0.001	1.18 (1.09–1.27)	<0.001	1.04 (0.95–1.14)	0.374
Glucose_max	1.00 (1.00–1.00)	0.850	1.00 (1.00–1.00)	0.827	1.07 (1.05–1.09)	<0.001	1.03 (1.00–1.05)	0.019
PT_max	1.03 (1.03–1.04)	<0.001	1.02 (1.01–1.02)	<0.001	1.03 (1.01–1.04)	<0.001	1.00 (1.00–1.02)	0.252
PTT_max	1.02 (1.01–1.02)	<0.001	1.01 (1.00–1.01)	<0.001	1.01 (1.01–1.02)	<0.001	1.00 (1.00–1.01)	0.529
WBC_min	1.01 (1.00–1.01)	0.001	1.01 (1.00–1.01)	0.003	1.01 (1.00–1.01)	0.030	1.01 (1.00–1.01)	0.027

*ICU, intensive care unit; PCO_2_, partial pressure of carbon dioxide; PO_2_, partial pressure of oxygen; PT, prothrombin time; PTT, partial thromboplastin time; WBC, white blood cells*.

In the PSM cohort, univariate and multivariate logistic regression analyses demonstrated that early severe thrombocytopenia was an independent risk factor for in-hospital mortality (Table 2).

### Platelet course and in-hospital mortality

Of the 450 patients with early severe thrombocytopenia in the original cohort, 368 were included in the platelet course study. A total of 114 patients (Group I) had a maximum platelet count of <50 × 10^9^/L within 72 h of ICU admission after the appearance of the minimum platelet count in the first 24 h. In addition, the maximum platelet count increased to 50–99 × 10^9^/L in 71 patients (Group II), 100–149 × 10^9^/L in 19 patients (Group III), and ≥150 × 10^9^/L in 164 patients (Group IV). Compared with Group I, the risk of in-hospital mortality was significantly lower in Group IV patients (OR, 0.301; 95% CI, 0.139–0.648; *P* = 0.002). Recovery of platelet count to normal levels in the short term is a protective factor for patient prognosis (Table 3).

**Table 3:** Regression analysis based on platelet recovery to assess the association between platelet course and in-hospital mortality in critically ill children with early severe thrombocytopenia.

	**Odds ratio**	**95% confidence interval**	***P*-value**
Group I (*n* = 114)	Ref		
Group II (*n* = 71)	1.316	0.643–2.695	0.452
Group III (*n* = 19)	1.115	0.337–3.691	0.858
Group IV (*n* = 164)	0.301	0.139–0.648	0.002

## Discussion

Our study showed that severe thrombocytopenia is common in pediatric ICUs, with an early incidence of ~10%. Logistic regression analysis demonstrated that early severe thrombocytopenia was an independent risk factor for in-hospital mortality in critically ill children. In addition, we studied the platelet course and found that platelet count recovery to normal levels in the short term may indicate a favorable prognosis.

The occurrence of severe thrombocytopenia varies by ICU due to the varying vulnerability of various patients to thrombocytopenia. It occurs in ~20% of medical ICU patients (12) and almost 45% of trauma ICU patients (13). The prognostic relevance of severe thrombocytopenia, a typical indication for coagulopathy in critical care units, is inconclusive. Although many researchers feel that severe thrombocytopenia increases the probability of mortality (14, 15), others argue that early severe thrombocytopenia is a symptom of a more serious underlying cause and is not an independent prognostic factor (16). In this study, we not only showed that early severe thrombocytopenia increases the risk of in-hospital mortality but also discovered that the platelet course is important.

Krishnan et al. found higher mortality in critically ill children admitted to the hospital with thrombocytopenia in the pediatric ICUs (17). However, the number of cases included was small, and the number of deaths in the subgroup analysis was <10. This conclusion is supported by extensive research. Using a large sample of data from the PICU database, our study demonstrated this conclusion. At the subsequent follow-up of the platelet course, we discovered that 44.6% of patients with early severe thrombocytopenia reverted to normal platelet counts within 3 days and had lower in-hospital mortality than patients who retained severe thrombocytopenia. A delayed increase in platelet counts could indicate that underlying organ damage has not been addressed, which is typically caused by prolonged sepsis (18, 19). The management of critically ill patients is a race against time, and every delay in the disease necessitates clinical attention and, if necessary, reevaluation of the condition.

The strengths of our study include a large amount of study data on critically ill children and the platelet course follow-up. Our study has some limitations. First, this was a retrospective single-center study, with most patients from China, resulting in an unavoidable potential bias. Therefore, the findings of this study must be confirmed through a multi-center prospective study. Second, in the platelet course study, we only examined the prognosis of patients with early severe thrombocytopenia, which does not represent the entire PICU patient population. Third, data on patients' in-hospital procedures and some laboratory results (e.g., blood urea nitrogen) were missing from the database; therefore, severity scores could not be assessed. Similarly, treatment measures, such as patient blood transfusion and plasma exchange, cannot be obtained from the database. Therefore, studies of the platelet course are considered to report all-cause platelet recovery and need to be interpreted with caution.

## Conclusions

Early severe thrombocytopenia is an independent risk factor for in-hospital mortality in critically ill children. Furthermore, in-hospital mortality was significantly reduced in children with early severe thrombocytopenia whose platelet count returned to normal levels in the short term.

## Data Availability Statement

The original contributions presented in the study are included in the article/supplementary material, further inquiries can be directed to the corresponding author.

## Ethics Statement

Ethical review and approval was not required for the study on human participants in accordance with the local legislation and institutional requirements. Written informed consent from the participants' legal guardian/next of kin was not required to participate in this study in accordance with the national legislation and the institutional requirements.

## Author contributions

YL extracted and analyzed the research data. YL and CR wrote the first draft of the manuscript. HG participated in data analysis and review of the final manuscript. All authors contributed to the article and approved the submitted version.

## Conflict of interest

The authors declare that the research was conducted in the absence of any commercial or financial relationships that could be construed as a potential conflict of interest.

## Publisher's note

All claims expressed in this article are solely those of the authors and do not necessarily represent those of their affiliated organizations, or those of the publisher, the editors and the reviewers. Any product that may be evaluated in this article, or claim that may be made by its manufacturer, is not guaranteed or endorsed by the publisher.
